# Linkage to care of HIV positive clients in a community based HIV counselling and testing programme: A success story of non-governmental organisations in a South African district

**DOI:** 10.1371/journal.pone.0210826

**Published:** 2019-01-22

**Authors:** Simukai Shamu, Jean Slabbert, Geoffrey Guloba, Dalene Blom, Sikhulile Khupakonke, Nomea Masihleho, Julius Kamera, Suzanne Johnson, Thato Farirai, Nkhensani Nkhwashu

**Affiliations:** 1 Foundation for Professional Development, Health Systems Strengthening Division, Pretoria, South Africa; 2 University of the Witwatersrand, School of Public Health, Johannesburg, South Africa; 3 USAID, Pretoria, South Africa; Johns Hopkins School of Public Health, UNITED STATES

## Abstract

**Introduction:**

Although current data projects South Africa potentially meeting the UN target to test 90% of all people living with HIV by 2020, linking them to HIV care remains a big challenge. In an effort to increase linkage to care (LTC) of HIV positive clients an innovative collaborative intervention between two non-governmental organisations was developed and implemented between 2016 and 2017. This paper investigated the outcome of this collaborative intervention.

**Methods:**

We used a mixed methods approach to assess the outcome of the innovative relationship. This was done by analysing routine programmatic quantitative data on LTC between 2015 and 2017 and qualitatively interviewing five programme managers, four programme implementers and five HIV positive clients on their perceived success/failure factors. Qualitative data were analysed using thematic content analysis while LTC rates were descriptively analysed. Two consultative meetings presented draft findings to programme managers (n = 7) and implementers (n = 10) for feedback, results verification and confirmation.

**Results:**

In 2015 cumulative LTC rate was 27% and it rose to 85% two years post-intervention in 2017. Six themes emerged as success factors at the health system and structural levels and these include: provision of client escort services, health facility human resource capacity strengthening, inter and intra-organisational teamwork, onsite LTC, facilitated and expedited jumping of queues and shifting administrative tasks to non-clinical staff to protect nurses’ time on ART initiation. These measures in turn ensured increased, affordable and swift ART initiation of clients while strengthening client support.

**Conclusions:**

We concluded that multi-faceted interventions that target both health system challenges including staff shortages, efficiencies, and extended facility opening times, and structural inadequacies, including client time and resource limitations due to poverty or nature of jobs, can help to increase LTC.

## Introduction

The introduction of highly active antiretroviral therapy (HAART) in 1996 has allowed people living with HIV (PLWH) to prevent opportunistic infections, reduce their infectiousness, live longer and have a better quality of life[1,2]. In order to attain these benefits one needs to be tested for HIV and linked to care[3]. The long process of linkage to care (LTC)[4], that includes referring to health facility, conducting CD4 count at facility, HIV staging before treatment including long procedures of negotiating and counselling to achieve readiness for enrolling for ART and long administrative paper trail has been immensely shortened by the introduction of the WHO guidelines on universal test and treat[5], which was translated into the UN’s 90-90-90- pragmatic goals[6] that South Africa subsequently adopted.

Substantial losses of HIV diagnosed clients at pre-ART stages have been observed in a meta-analysis of studies conducted in Sub-Saharan Africa. Only a median of 44% (range 31%–68%) of all the people tested were initiated on treatment within 12 months[7]. Although South Africa seems to do well with the first 90 UN target (testing 90% of all people living with HIV), the second 90 target (initiating 90% of all HIV positive people on treatment) has proved difficult to meet[8]. Between 31% and 80% of people testing HIV positive were reported linked to care in South African settings[3,7,9–15]. Although earlier studies were conducted with facility-tested clients[16], almost all studies reported above were community based programmes. With as low linkage to care as 31% reported in some studies[7] South Africa cannot meet the second 90 UN target while lessons must be drawn from programmes achieving high LTC rates.

A number of studies have suggested a plethora of barriers to LTC. A systematic review of barriers and facilitators to LTC found psychosocial, economic, health systems and medical barriers in 25 articles in Southern and Eastern Africa with most of the studies from South Africa. The most cited barriers were transport costs and long distance from home to the nearest referral health facility, while stigma and fear of HIV status disclosure constituted the second most commonly cited barriers[17,18]. Other barriers include staff shortages, long waiting times, rural clinics with low staff levels[19], fear of drug side effects or shortages[10,17], younger age[20] and the need to take time off work[17]. Some of the factors associated with lower LTC rates include being male[14,20,21], belonging to a low socioeconomic status[21], a history of TB[18], deferring CD4 count[22], and having a low CD4 count[11,21,23,24]. Factors associated with high LTC were experiencing three or more depression symptoms, being a caregiver for four or more people, living with someone attending HIV care and knowing someone who died of HIV/AIDS[10,25]. Individuals with more barriers or risk factors were more likely to be lost to care initiation than those without the risk factors[18]. Although it is important to assess individual level factors, which were found to be mostly associated with low LTC rates, fewer studies assessed health system and structural interventions and yet these are equally important. Based on the assessment of the barriers to LTC it is clear that innovative and multifaceted interventions are needed to address these shortcomings of current programmes at the level of the health system, family/partnership, community and individual to increase LTC rates.

To date, a number of strategies have been implemented to improve linkage rates. In a qualitative review of strategies to improve LTC, the most common and successful intervention found was the use of an active coordinator to help LTC while others that include, offering HIV care information, education and counselling, provider accompanying clients to medical appointments and helping with appointment coordination and home visits[15,26–31] had notable successes. However, these interventions were almost exclusively focussed on breaking the individual barriers while leaving out health system and structural factors. Only a few studies assessed structural factors[19,26]. For example, health system facilitated LTC yielded as high as 95% facility visits for LTC and 75% ART initiation in community-based HIV testing centres compared to only about a quarter (26%) when there was no facilitated LTC[32]. Incentivised LTC and a call centre use have been reported to achieve high LTC rates compared to simple referral[32] although incentives need to be scalable and sustainable in real life. The use of technology such as point of care to fast track processes for enrolment have also shown an increased LTC in South Africa by over 300%, while reducing time to LTC [33,34] although other studies did not find a difference with the standard process^13^.

Despite an improvement in LTC rates brought about by these interventions, clients are still lost between HIV diagnosis and ART enrolment. In order to meet the second 90 UN target for South Africa, the Foundation for Professional Development (FPD)’s community-based HIV counselling and testing (CBCT) programme offered an opportunity to innovatively implement facilitated LTC in one high HIV burden district. The aim of this paper is to describe FPD’s facilitated LTC programme and highlight its outcomes in King Cetshwayo district, in KwaZulu Natal province, between 2016 and 2017. The paper documents the extent to which FPD’s CBCT programme addressed barriers mostly cited in South Africa to improve LTC rates.

## Methods

The study received ethical clearance from the Foundation for Professional Development Health Research Ethics Committee.

### The collaboration and intervention

FPD conducted CBCT in three sub districts covering urban, peri-urban and rural communities of King Cetshwayo district, KwaZulu-Natal province. The programme employed at least 40 employees with varying level of efforts in the study district. The CBCT’s LTC rate was the litmus test for programme success in terms of its contribution to the second 90 UN target for South Africa. LTC was traditionally done by simply referring a client to a health facility and later verify by visiting, reviewing and consolidating the health facility referral data sources including the national monitoring and evaluation database for ART, CD4 blood books, TB suspect register, and patient files using the referral list. Clients not yet linked to care were then reminded by phone calls, short message service or home visits to visit the health facility. This method did not yield much LTC—as low as 26.6% HIV positive clients were linked to care between October 2015 and September 2016. A number of challenges contributed to this low linkage to care and these include that personnel responsible for linking clients to care (described as linkage tracers) had to provide transport for themselves and clients who did not have the money to go to the health facility and were reimbursed by the programme at the end of the month. Other challenges include that HIV positive clients joined long queues and took up to a full day to initiate ART at the clinic leading to new clients avoiding visiting the clinics. CBCT clients joined the queues like any other patients and complained risking losing income while attending a clinic for long hours. The recently HIV tested clients also felt it was stigmatising to be seen joining queues at health facilities as people who knew them easily suspected them to have tested HIV positive. In addition counsellors were not easily accepted at the clinics when they wanted to review linkage to care data from the clinics. In order to increase LTC FPD designed and implemented an innovative programme of task shifting LTC services to an external service provider, BroadReach, through a collaborative arrangement between October 2016 and September 2017. FPD engaged BroadReach as a District Support Partner to assist with linking their HIV positive clients to care. BroadReach, a non-governmental organisation, whose mission is to improve access to healthcare through data use and technological advancements, operated in the province to support the South African Department of Health at district level with LTC. In the collaboration, FPD conducted HIV testing and supplied BroadReach with a list of people who tested HIV positive to link them to care at the health facilities through BroadReach’s linkage to care nurses based at the health facility. In addition, the nurse visited, escorted and assisted clients in the community, if they were unable to go to the facilities because of time or financial constraints. The FPD-BroadReach collaboration introduced patient escorts to fast track LTC process and its verification. This was done based on client needs and potential barriers to LTC. Clients were offered referral escorts at the time of HIV testing or at follow up visits to navigate the referral system and access the required care. The new approach used programme vehicles to escort clients to be linked to care while staff also conducted other duties such as mobilising clients for HIV testing or conducting door-to-door HIV testing.

This paper is based on a mixed methods approach that combined both qualitative and quantitative data collection and analysis methods [35]. Through the mixed methods approach, the quantitative analysis illustrated the outcomes of the LTC approach over two years, while the qualitative methods described its outcomes.

#### Routine quantitative programme data

We utilised routine programme data collected between October 2015 and September 2017 on HIV clients’ LTC in King Cetshwayo district. The methodology for the CBCT programme has been reported elsewhere (Kuwanda et al forthcoming). Assessing two years’ CBCT data helped to trace trends of LTC rates in the district before and after the innovations. The database contained demographic (age, gender, residence town) and clinical data (ART initiation, clinic visits and dates). LTC was defined as confirmed HIV positive people successfully referred to and visiting health facility/professional for ART enrolment.[36] We therefore presented the LTC rate as a percentage of all the people tested positive and referred for care.

#### Qualitative data

After analysing the LTC rates and trends, we assessed the LTC outcomes. The research team purposively selected and interviewed fifteen key informants using an interview guide; these are five (5) programme managers (monitoring and evaluation specialists, clinical nurses, and programme specialists) and five programme implementers (linkage nurses, linkage tracers, counsellors, and data capturers) and five clients linked to care. Purposive sampling allowed enrolling participants believed or known to have the information required to answer the study objectives. Formal interviews were conducted face to face or telephonically if it was not possible to schedule a face to face interview with the participant. They were asked to reflect on the documented process, LTC outcomes and their perceived ideas regarding the programme success and challenges. Five adult clients—two male and three female who were linked to care by nurses employed by a non-governmental organisation, BroadReach, were purposefully selected from the programme database and interviewed on the process used to link them to care and their experiences with expediting, escorting, following up and enrolling for ART. The interviews with programme managers and implementers were conducted in English while clients were interviewed in isiZulu. Monthly minutes of the project meetings were reviewed and referred to in order to understand and confirm how linkage to care processes were implemented and reviewed. After all interviews and data reviews were done, the researchers convened a meeting with programme managers (n = 7) and implementers (n = 10) to verify and confirm the written findings. All formal interviews were audio recorded while notes were taken in consultations to verify data.

The transcribed and translated interview data were cleaned and confirmed for accuracy prior to analysis. In order to check transcription accuracy and reliability, an independent transcriber randomly selected and examined five percent of the transcriptions. These checked transcriptions were back-translated and compared with the original script to detect and correct any deviations. We aimed at having the transcriptions re-transcribed by another qualified transcriber if more than permissible discrepancies were observed [37], but did not do so as there were only allowable discrepancies. Data were ordered into codes that formed categories, which were all ordered into a theme if they referred to a similar success or challenge. Two researchers separately analysed the transcripts using pre-defined codes and categories and later discussed these to confirm if they were aligning or diverging[37]. New codes emerged from the data and these supplemented the pre-defined codes. Two researchers analysed the coded transcripts to identify themes as an iterative process through a constant comparison approach[38,39]. Disagreements were resolved by mutual agreement by the two researchers or by consulting a third independent researcher. The themes were then presented to other researchers including programme implementers and managers for discussion and confirmation of the outcomes. The study received ethical clearance from the FPD Health Research Ethics Committee.

## Results

From the quantitative data, cumulative LTC rate rose from a low of 27% (1 407 linked to care out of 5 292 HIV positive clients) in 2015 to 85% in the year 2016/2017 (4127 linked to care out of 4876 HIV positive clients). LTC continued to rise in the second year of implementation (2017/2018) to 90% (3 407 clients linked to care among the 3 766 HIV positive clients). From the qualitative data six themes emerged as LTC success factors at the health system and structural levels leading to increased, affordable and swift ART initiation of clients while strengthening client support. The themes are described below.

### Provision of client escort services

As described earlier LTC experienced challenges including having to navigate through transport challenges and long queues at health facilities. The provision of transportation from home to the referral facility for LTC contributed towards improved linkage rates. With prior request made to the client that there was a possibility of the same vehicle carrying at least one more client to be linked to care at the same clinic with them, linkage tracers were able to escort more than one clients in one vehicle at the same time. Seeking client consent was done to avoid unintended, unwanted and indirect disclosure of one’s HIV status to fellow HIV positive clients on the same vehicle. It also provided an opportunity for peer support and promoted adherence to ART since clients in the same community could encourage each other and work together for pill refill. Thus the use of vehicles had multiple roles—HTS, LTC, peer client support, promoting adherence and so increasing cost effectiveness. In situations where HIV testing was conducted through the mobile modality and when mobile stations were set up close to the health facilities linkage tracers would easily walk the clients to the health facility for ART initiation instead of simply referring them for LTC. It was also confirmed in technical meetings that children really needed escorts to be linked to care. It was after escorts were implemented that many children were linked to care. One project implementer said that:

“*Discussions to link children helped so much as it was noted that children can better be linked with assistance of the district Service Provider through escorting since they [children] cannot*, *on their own*, *make decisions neither can they take themselves to the clinic*. *By convincing parents in denial*, *children were escorted with parents’ permission and presence for linkage*.*”*

In a boardroom, a technical manager remarked in agreement with the quantitative data and qualitative data from the counsellors: *“so we managed to turn the red to green by targeting the [children aged] 1–14 years*. Another programme manager in a different meeting remarked, *“The children (1–14) were always red*, *so we turned them green through home visits and transportation to the clinics”*.

The reference to colours described the monitoring and evaluation excel sheets that were colour coded to show extremely low linkage rates in red (below 50%) and over 80% linkage rates which were coloured green. Although children between 1 and 14 were few, it was initially difficult to link them to care but with these targeted escorts it was made possible.

### Facilitated jumping of queues

To address the problem of queuing for ART initiation for too long a strategy to expedite ART initiation for clients at the facilities was implemented. BroadReach enabled expediting ART initiation for clients at the health facilities by swiftly attending to referred CBCT clients. This increased the number of people linked to care as clients could no longer wait for too long before being initiated on treatment. One BroadReach nurse specifically initiated CBCT clients on ART while the facility nurses continued with their daily duties as explained by the District CBCT coordinator:

*“…without fast-tracking*, *clients used to spend 3–4 hours waiting for consultation with the DoH (Department of Health) nurse*. *Sometimes (just) before your (client’s) turn*, *the nurse would go for lunch or tea break*, *what would you do*? *… so I approached our District Service Provider and said*: *is it possible not to keep our clients waiting*? *Instead open a clinical chart (patient record) for them then the District Service Provider nurse will initiate because we can’t expect the Department of Health nurse to stop consulting other people…”* District CBCT Coordinator. It was also reiterated by a male client that *“joining*, *not starting the queue yourself*, *at every point—to get a clinic registration*, *to open a medical book*, *etc etc*, *then another queue for the initial pills was very problematic for many clients not escorted”*.

With the BroadReach nurse assistance in initiating care for CBCT clients waiting time was significantly reduced as the nurse who initiated clients reported that *“…clients now spend an hour at most in the clinic for ART initiation…”*

### Task shifting of administrative duties to linkage tracers at ART initiation

Initially, counsellors were not easily accepted at the clinics when they wanted to review linkage to care data from the clinics. After a relationship with BroadReach was established they were then accepted and could review data and could even conduct some administrative duties at the clinic on behalf of the nurse. When clients were escorted to the facility by the linkage tracer for ART initiation the linkage tracer expedited the procedures by assisting with administrative tasks including to register the client at the facility, accompany them for vital sign assessment, register the client in blood book and open a client clinical chart. When all the administrative tasks were completed, the client was then handed over to the District Service Provider nurse for ART initiation. The shifting of administrative duties to the linkage tracer at the clinic reduced the clients’ waiting time to only 30–40 minutes from more than three hours and allowed the District Service Provider nurse to concentrate on initiating treatment to clients. Shifting of tasks to the District Service Provider nurses from the health facility nurses who were already overwhelmed by managing clients with HIV or non-HIV issues was reiterated by one staff as contributing towards real time access to data relevant for LTC as follows:

“Without a District Service Provider you find that someone was linked to care but not captured [onto the system]. So with the District Service Provider nurse who has quick access to the information database you have real time data on who is linked to care or not and this information helps to speed up LTC”.

Shifting administrative tasks therefore contributed towards expediting the linkage process thereby creating a motivating factor for linkage to care for clients.

### On-site linkage to care

The relationship between FPD and BroadReach helped to increase LTC by initiating HIV positive clients on-site at the point of HIV testing as the District Service Provider could travel with the CBCT team to avoid loss of HIV positive clients. Initiating care to clients on site (home, work or any convenient place in the community) cut transport costs of the clients for LTC, it also brought access to treatment to those who had time constraints to travel to the referral facility due to work or other commitments. For example, the District Service Provider nurse was able to accompany counsellors identifying and testing members of an infected person’s sexual network or family on many occasions and would initiate clients on ART immediately after testing. The nurse mobility enabled providing ART initiation services outside of the traditional facility opening hours. Those clients with time constraints were therefore covered by the extended hours for ART initiation by the District Service Provider nurse including working extended hours during week days, during weekends and public holidays when the facilities were closed. The mobility of the District Service Provider nurse facilitated the seamless provision of HIV testing and LTC services.

On ART initiation, the District Service Provider nurse gave medication and a return date for further management while pill refill was also organised. The nurse also registered the client at the client’s preferred and convenient health facility and then sent a message to the client’s mobile phone notifying them of their patient or file number. The referral form was stamped and kept in the client’s file and a copy was submitted to FPD for linkage confirmation. The onsite process enabled same-day and immediate LTC thereby reducing clients being lost in the process of referral for LTC.

### Intra-and-inter NGO teamwork

One of the success factors was the team work among FPD’s CBCT team members with BroadReach nurses as well as among FPD team members themselves. The CBCT team members including the District Service Provider nurse planted into the CBCT team worked together well despite each one having individual responsibilities e.g. the counsellor tested clients, the linkage tracer traced clients for LTC while the BroadReach nurse initiated clients on ART and the driver/counsellor drove the team and also conducted HTS. Despite these designated roles, ultimate linkage of clients to care was made every team member’s responsibility. One linkage tracer reported:

“…our coordinator told us that LTC is everyone’s business and that if patients are not linked to care then it means CBCT is failing. So even the HTC counsellor sometimes links patients to care…everyone is involved.

Team members constructively shared successes as well as failures without blaming each other. At the office a data capturer posted weekly statistics showing uptake of HTS, positivity and LTC rates on a publicly observable noticeboard for all CBCT staff to see. This helped the team to keep track of and celebrate progress while seeking solutions to challenges including when figures showed that the team was behind on targets. Team members felt motivated and committed to achieving excellence. Constant informal meetings and reference to statistics updated on the office’s notice board kept team members updated on programme progress towards meeting targets. One linkage tracer narrated how the noticeboard statistic motivated them to achieve their targets as follows:

*“When counsellors come and look at the board*, *they can say*, *last week we found say five HIV positive clients but only two were linked… what happened to the others*?....*”* Linkage tracer.

One project manager shared the same sentiments saying, “*Linkage is a joint activity…we jointly own the positivity and link the positive clients together”* with BroadReach. Another manager reiterated the strong relationship and interaction between BroadReach and FPD saying, *“…even after church the District Service Provider nurse calls us and checks ‘is there a person for me to link today’”*. This shows a sense of duty and responsibility by team members in the FPD-BroadReach partnership.

Such engaging with targets was instrumental in motivating the team for their work. The FPD team also felt letting down the BroadReach nurses if they did not provide them with the necessary information. The fact that the FPD team travelled to the testing sites with external people (BroadReach) whose time they did not want to waste with a small pool of clients to link to care pushed them to be on target with testing while also the BroadReach nurse felt the need to always ensure that linkage targets were met.

### Challenges encountered during implementation

Despite these successes, there were challenges that faced the programme’s LTC process including capturing incorrect follow up contact details during HTS. The BroadReach nurses’ success in follow up was hinged on successfully contacting the people to be linked and hence the FPD team had to ensure this was done. To address this challenge counsellors buzzed the clients’ mobile phones at the time of capturing the numbers to test if the numbers were correct and functioning. While onsite LTC conducted immediately after testing prevented loss to care it did not provide adequate time to a client to process information about being an HIV positive person and the HIV treatment before they were linked to care. One client remarked, “*I did not visit the clinic or start treatment immediately because I needed to discuss this [HIV positive status] with my family before rushing to the clinic*”. Some of the challenges include that some clients resisted LTC, for example, by not responding to their mobile phone calls. One counsellor recalled, *“Home visits for linkage was strengthened after realising that some clients did not respond to their mobile phones*, *so we visited them to get them to the clinic to link them”*. Another challenge was that some health officials at facilities did not fully recognise the District Service Provider nurse and linkage tracers’ support to fast tracking CBCT clients. This was being addressed at the time of data collection for this paper.

## Discussion

The study found high levels of linkage to care rates achieved through multiple facility and community based interventions at different levels by a partnership of non-governmental organisations. Six success factors found were provision of client escort services, human resource capacity strengthening at the health facility, inter and intra-organisational teamwork, onsite linkage to care, facilitated and expedited jumping of queues and shifting administrative tasks to non-clinical staff while ring-fencing nurses’ time on ART initiation. These measures not only increased linkage to care but also ensured less costly but fast client ART initiation while strengthening peer client support and reducing pressure of work among existing health facility staff.

Results of the study show the impact of a multifaceted intervention that targets barriers to linkage at multiple levels especially at the level of the health system and the structural levels. Despite some studies seeing a less beneficial relationship between NGOs and the public health sector[40] our study demonstrated non-governmental organisations’ critical role in strengthening public health systems[41] by providing additional health care nurses to increase and strengthen existing but overwhelmed health facility staff for ART initiation. Since the District Service Provider nurses were located in a public health facility there was a high level of private-public sector integration and thereby avoiding running parallel health system for HIV which has limitations on sustainability once donor funding ceases[40]. The study therefore provides a case study of a successful partnership between non-governmental organisations as well as collaboration with the public health sector to achieve the health system goals.

Our results further demonstrate the importance of task shifting from state public health nurses to NGO nurses and facility administrative duties from nurses to linkage tracers resulting in increased linkage to care. This protected nurses’ time to conduct more professional tasks while linkage tracers conducted administrative duties. As reported in Sub-Saharan Africa[42] task shifting helped to expand HIV services to less resourced communities with good quality at no cost to clients and affordable costs to health facilities. Our study provided another model of task shifting that included shifting facility tasks to the private sector including conducting administrative duties which consumed health facility nurse time resulting in long queues for new ART clients. Shifting the administrative tasks from the public facility nurse to the NGO linkage tracer gave the nurse more time to initiate clients with HAART which resulted in reducing long waiting times at the clinic which further enabled attracting more clients for linkage to care as people realised that queues were shorter. It is also important to note that our study found tasks of care initiation shifted from the clinic to the community where people live and work. By so doing, health facilities were decongested and also making less work for the public health facility.

The study also demonstrated that in a resource limited society, where the majority of the community members do not earn an income bringing community access to health by covering or removing transport costs from the individual helps to increase uptake of services. Transport barriers have often been cited as major inhibiting factors for linkage to care to happen[17,18]. The escorting of clients to the facility and ART initiation at the site cut transport costs and ensured clients could not be lost to care by reducing time between testing and ART initiation. Recent studies providing conditional incentives for transport in resource constrained communities have shown greater and quicker uptake of services in those provided with transport incentives than people with no transport incentives[43]. In any intervention that involves incentives, the question of sustainability of such services requires that such services be scalable. Although a costing study is required we have reason to believe that our model was less costly because the sharing of transport facilities by the HIV testing and linkage team was able to cut costs as one car was provided per team for a wide range of activities in one community since the District Service Provider nurse planned visits within one locality where the HIV testing team was also working. Nevertheless, a costing study is required to compare different models including escorting on car, providing bus fare to clients, self-referral, on-site linkage vis a- vis time and cost saved, lives saved and disability adjusted life years.

Confidentiality of HIV test results is an important human right and ethical issue for the success of CBCT programming[44]. In our study we found extra care being taken by LTC staff to ensure that clients were pre-warned and were requested to consent to be transported in one car for linkage at a preferred referral health facility or community place as a matter of improving efficiency. Seeking consent and pre-warning clients showed responsiveness of the staff in providing quality and ethical service to PLWH and being mindful of possible violence as a result of indirect HIV status disclosure in a setting where partner violence is common[45]. This strategy enabled clients to trust their health providers, prepare themselves, organise peer support in adherence and it also helped to ensure that clients could plan pill refill together. Such factors are protective for ART adherence.

We also reported challenges of some tension between public health facility staff and NGO staff. Some studies warn about the inescapable tension between facility nurses and NGO community health workers around unclear roles and hierarchies within the clinic[40]. Similar initial challenges which however get naturally solved when programme staff meet and discuss way forward in routine monthly or weekly meetings were also reported in our study. Such challenges are expected and require addressing at the onset by drawing memoranda of understanding with clear duties, boundaries and hierarchies for smooth operations.

Our study has limitations which must be understood to help interpreting the results. Firstly, more clients could have been consulted to describe programme outcomes, for example recruiting more clients who were linked to care through different ways or those not linked to care would have increased the richness of the qualitative data including challenges of linkage to care. The quantitative data used in this analysis were routine data that was not collected for the purpose of this paper. Many variables such as individual factors associated with the programme success could have been assessed if the study was planned prospectively. Also, a comparison with other districts that were offering the standard LTC facilities would have improved the analysis. Nevertheless, the before and after intervention analysis was instrumental in showing the effect of the intervention on LTC. We believe that such a design is strong in evaluating intervention impact. In addition being a mixed methods study, the study was able to utilise the strengths of a qualitative and quantitative study and as such able to explain qualitatively the increase in rates of linkage to care as observed by programme managers and implementers. We were able to mix methods and verify information provided in quantitative data through interviews and lastly through consultative meetings which confirmed our themes on success factors and challenges the programme faced.

## Conclusions

We reported a success story of linkage to care in a low resource urban, peri-urban, and rural community as facilitated by partnerships between two private institutions in public health provision and strengthening. Interventions that target both health system challenges including staff shortages, staff efficiencies and extending service opening times, and structural inadequacies including client time and resource limitations due to poverty or nature of jobs can help to increase linkage to care. We recommend multiple interventions to increase linkage to care in community-based HIV counselling and testing programmes. We also recommend building strong partnerships for the success of the UN’s 90-90-90 HIV targets for South Africa.

## Supporting information

S1 File Supporting information. (DOCX)
