# Supplementary material for: Linkage to care of HIV positive clients in a community based HIV counselling and testing programme: A success story of non-governmental organisations in a South African district
Source: PLoS One. 2019 Jan 22;14(1):e0210826. doi: 10.1371/journal.pone.0210826 (PMC6342293; doi:10.1371/journal.pone.0210826)
Supplement: S1 File — (DOCX) [file pone.0210826.s001.docx]

**Qualitative data – Linkage to care key themes**

**Escort services**

- Cars to transport clients plus tracer
- When carrying two clients – avoid unintended disclosure of HIV status – prior request
- Use of statistics on noticeboards to increase monitoring

One project implementer said that:

“*Discussions to link children helped so much as it was noted that children can better be linked with assistance of the district Service Provider through escorting since they [children] cannot, on their own, make decisions neither can they take themselves to the clinic. By convincing parents in denial, children were escorted with parents’ permission and presence for linkage.”*

In a boardroom, a technical manager remarked in agreement with the quantitative data and qualitative data from the counsellors*: “so we managed to turn the red to green by targeting the [children aged] 1-14 years.* Another programme manager in a different meeting remarked, *“The children (1-14) were always red, so we turned them green through home visits and transportation to the clinics”.*

**Fast tracking queues**

- CBCT teams assisting with non-clinical aspects
- Clients happy to spend less time
- How collaboration was initiated
- What prompted collaboration
- Peace in collaborations

**Quotes**

*“…without fast-tracking, clients used to spend 3-4 hours waiting for consultation with the DoH (Department of Health) nurse. Sometimes (just) before your (client’s) turn, the nurse would go for lunch or tea break, what would you do? … So I approached our District Service Provider and said: is it possible not to keep our clients waiting? Instead open a clinical chart (patient record) for them then the District Service Provider nurse will initiate because we can’t expect the Department of Health nurse to stop consulting other people…”* District CBCT Coordinator. It was also reiterated by a male client that *“joining, not starting the queue yourself, at every point – to get a clinic registration, to open a medical book, etc etc, then another queue for the initial pills was very problematic for many clients not escorted”.*

With the BroadReach nurse assistance in initiating care for CBCT clients waiting time was significantly reduced as the nurse who initiated clients reported that *“…clients now spend an hour at most in the clinic for ART initiation…”*

**FPD-B/reach teamwork**

- FPD teams worked together for common good
- FPD-Broadreach collaborated together well in the field and at clinic
- “…our coordinator told us that LTC is everyone’s business and that if patients are not linked to care then it means CBCT is failing. So even the HTC counsellor sometimes links patients to care…everyone is involved.
- “ When counsellors come and look at the board, they can say, last week we found say five HIV positive clients but only two were linked… what happened to the others?....” Linkage tracer.
- Project manager: “Linkage is a joint activity…we jointly own the positivity and link the positive clients together” with BroadReach. Another manager reiterated the strong relationship and interaction between BroadReach and FPD saying, “…even after church the DSP District Service Provider nurse calls us and checks ‘is there a person for me to link today’”. This shows a sense of duty and responsibility by team members in the FPD-BroadReach partnership.

**Onsite linkage**

- Linking clients at the site – meaning nurse travels with CBCT team to the field. Initiates clients at the home or community and carries documents to clinic for storage…
- Makes appointments for follow up
- Cut transport costs for clients

**Task shifting**

*Saved time from 8 hours to 30-40 mins*

*“Without a District Service Provider you find that someone was linked to care but not captured [onto the system]. So with the District Service Provider nurse who has quick access to the information database you have real time data on who is linked to care or not and this information helps to speed up LTC”.*

**Challenges**:

- Challenges of onsite linkage to care – no time to digest meaning of positive results
- Mobile phones not reachable – so had to test them in field immediately
- Tracers not fully recognised by facility nurses

Quotes:

, “*I did not visit the clinic or start treatment immediately because I needed to discuss this [HIV positive status] with my family before rushing to the clinic*”. Some of the challenges include that some clients resisted LTC, for example, by not responding to their mobile phone calls. One counsellor recalled, *“Home visits for linkage was strengthened after realising that some clients did not respond to their mobile phones, so we visited them to get them to the clinic to link them”.*
